# Clinical and radiologic outcomes after a modified bone plug technique with anatomical meniscal root reinsertion for meniscal allograft transplantation and a minimum 18-month follow-up

**DOI:** 10.1186/s13018-018-0776-3

**Published:** 2018-04-24

**Authors:** Shiyou Ren, Xintao Zhang, Tian You, Xiaocheng Jiang, Dadi Jin, Wentao Zhang

**Affiliations:** 1 Department of Sports Medicine and Rehabilitation, Peking University Shenzhen Hospital, 1120 Lianhua Road, Futian District, Shenzhen, 518036 Guangdong China; 2 Department of Orthopaedics, The Third Affiliated Hospital of Southern Medical University, Guangzhou, Guangdong China

**Keywords:** Meniscal allograft transplantation, Modified bone plugs, Knee, Arthroscopy

## Abstract

**Objective:**

To evaluate the clinical and radiologic outcomes of meniscal allograft transplantation (MAT) using a modified bone plug technique.

**Methods:**

We conducted a retrospective single-center study of 73 patients who underwent MAT between January 2007 and December 2013. The International Knee Documentation Committee (IKDC) score, Lysholm score, Tegner score, visual analogue scale (VAS), and physical examinations were retrospectively reviewed to measure clinical outcomes after MAT, and questionnaires regarding activity and factors were analyzed. Magnetic resonance imaging (MRI) was used to assess the cartilage status and meniscal extrusion.

**Results:**

The mean follow-up was 37 months for 61 patients (65 knees), and 12 patients were lost to follow-up. The mean meniscal extrusion was 3.39 ± 0.90 mm, the relative percentage of extrusion (RPE) was 34.82% ± 12.71%, and arthrosis progression was observed in 8 of 61 cases (13.1%). The mean results for VAS, IKDC, and Lysholm scores were significantly improved after MAT (*P* < 0.05), but there were no significant differences in the range of motion or Tegner score (*P* > 0.05). Thirty-eight (62.3%) patients were able to return to their previous level of activity, and 23 (37.7%) patients reached a mean 76.7% of the previous level of activity. Of the 23 patients reporting a decrease in activity, 10 reported a fear of reinjury as the primary factor limiting activity. The patient satisfaction rate in the study was 78.7%.

**Conclusion:**

Our modified bone plug method with anatomical meniscal root reinsertion was an effective surgical method, and the majority of active patients with meniscal disorders returned to preinjury levels of activity.

## Introduction

The meniscus plays an essential role in the function and biomechanics of the knee joint, providing an even load distribution across the joint, thereby decreasing peak contact forces on the tibiofemoral articular cartilage [1]. Meniscus tears are one of the most common injuries in sports medicine and may result either from acute knee trauma or through degenerative processes. The management of meniscal tears is varied and often dependent on the severity of the injury, including nonoperative treatment, meniscectomy, repair, and transplantation [2]. In cases of irreparable meniscus tears or meniscal deficiency after meniscectomy, there may be considerable pain and there is a high risk of developing degenerative disease of the knee joint over time. How to restore the function of the meniscus to induce an early regenerative progress has increasingly been recognized as a clinical challenge. Since the meniscal transplantation procedure was first described by Milachowski et al. in 1984 [3], meniscal allograft transplantation (MAT) has become a viable option in meniscus-deficient patients.

However, MAT remains a controversial treatment for meniscus-deficient patients [4] because of its underlying conditions of uncertainty, such as whether living-donor tissue is required for optimal attachment, the long-term outcomes, whether it can delay the degenerative progress, and whether movement should be restricted postoperatively. Fortunately, an increasing number of authors have attempted to develop this procedure and have reported good results. However, the use of bone plugs is still controversial. Certain studies have achieved good clinical and biomechanical results with bone plugs [5–7]. However, good clinical results using only suture fixation have also been described [8, 9]. Furthermore, it is essential to ensure a precise size match between the graft and the host in techniques involving bone plugs, which can increase the risk of cartilage degeneration and incorrect positioning [7]. It can reduce the morbidity of surgery with suture fixation and can be performed under arthroscopy. In contrast, it is technically easier to perform fixation with soft tissue alone, but research has shown that the load distribution is superior when the allograft is secured with bone [10, 11]. Therefore, we used an arthroscopic double tibial tunnel technique for MAT with new modified bone plugs to ensure initial fixation and sound bone-to-bone healing in a consecutive series of symptomatic patients with a previous total or subtotal meniscectomy [12]. The purpose of this study was to evaluate the clinical and radiologic outcomes of MAT using a new modified bone plug technique with anatomical meniscal root reinsertion.

## Patients and methods

### Study overview

This study and procedure were approved by our institutional review board. From January 2007 to December 2013, 73 consecutive patients underwent MAT. Of these, 61 patients (36 men and 25 women) with a mean age of 32.3 years were followed up for more than 18 months and were enrolled in this study. The follow-up rate was 83.6%, and the mean follow-up duration was 31.0 months (18–80 months).

Indications for surgery included irreparable tears diagnosed preoperatively and during arthroscopy or persistent symptoms after meniscectomy, normal alignment or correction-to-normal alignment, and a stable ligamentous knee condition or correction to a stable ligamentous knee condition. Five out of 61 patients underwent immediate MAT without any chondral injury or symptoms after meniscectomy, and the remaining patients underwent delayed MAT with symptoms of pain or decreased range of motion (ROM) after meniscectomy.

Preoperative radiographic assessment was performed on all patients including the following: weight bearing anteroposterior and 3D CT of the knee, a lower extremity examination and MRI. The irreparable meniscal tears were diagnosed by MRI preoperatively and confirmed by arthroscopy. The occurrence and degree of osteochondral injury were assessed by MRI using the Outerbridge grade. Meniscal deficiency was confirmed by a history of arthroscopic meniscectomy and MRI.

There were 21 left knees and 35 right knees with lateral MAT, 3 knees with medial and lateral MAT and 6 right knees with medial MAT. Four patients underwent lateral MAT of both knees. Not all of the patients received meniscal transplantation alone. Some patients were found to have concomitant anterior cruciate ligament (ACL) injuries, cartilage injury, MCL injuries, or contralateral meniscus injury in the same knee; therefore, additional orthopedic procedures— medial collateral ligament reconstruction (MCLR), anterior cruciate ligament reconstruction (ACLR), and MF—were performed in some cases, and for one patient, osteotomy was performed to address coronal malalignment of the lower limbs. Additional procedures performed at the time of MAT are included in Table 1. The mean time from the total meniscectomy to the secondary MAT was 36.6 weeks. Three patients underwent a lateral MAT, combined medial MAT and ACLR at the same time. These procedures showed the advantages of the new technique in that little space is required to fix the anterior or posterior horns of the meniscus, and both lateral and medial MATs were secured on the tibial simultaneously without damaging the tibial insertion of the PCL or encroaching on the sites for tibial tunnels of ACLR.

**Table 1:** Combined operation with meniscal allograft transplantation in all 61 cases

	ACLR	Microfx	OT	MCLR	CMSK	ORIF
L-MAT	7	9	2	1	1 (MAT)	1
M-MAT	3	2	0	0	1 (repair)	0
L/M-MAT	3	0	0	0	0	0

### Surgical procedure

**Fig. 1:**
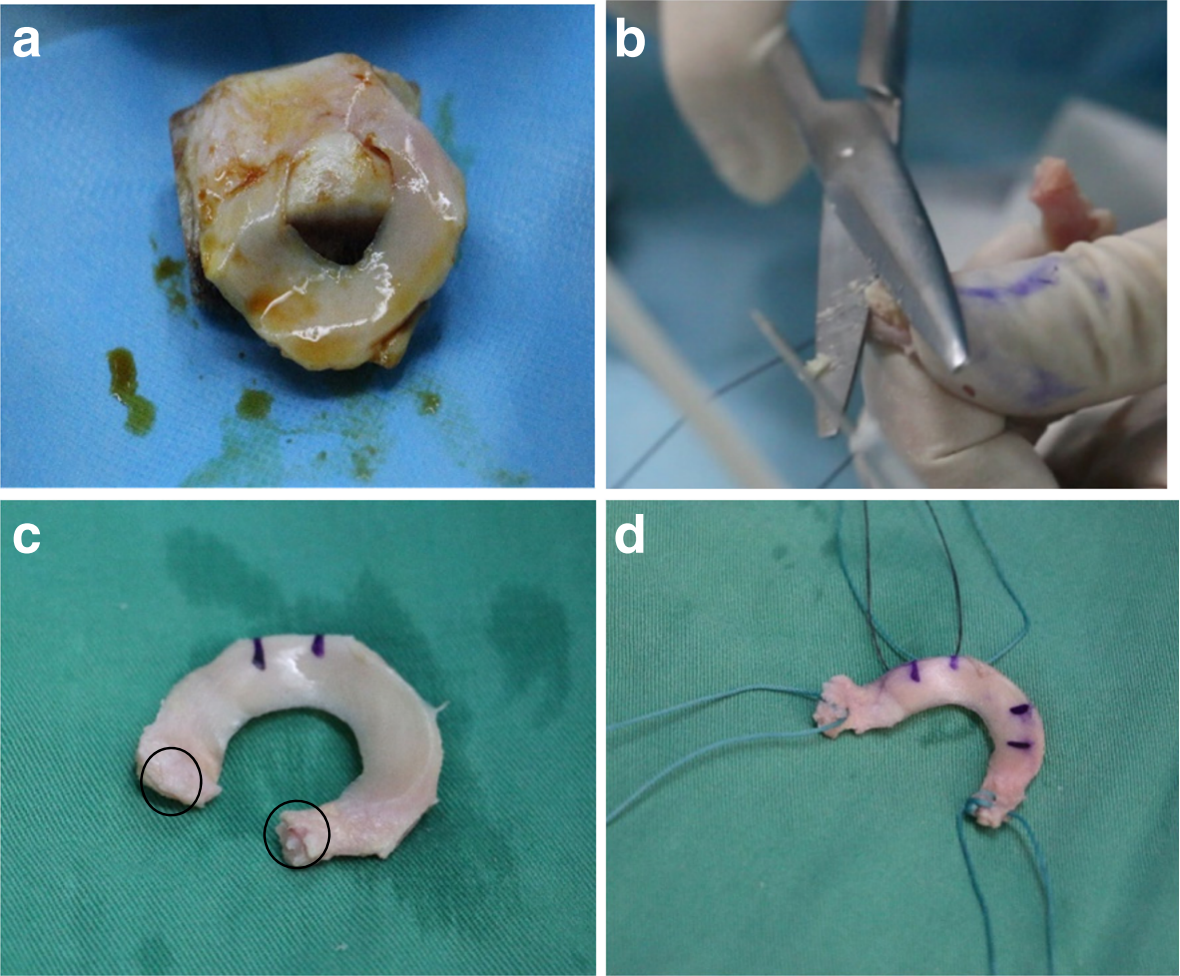
Trimmed meniscal allograft. **a** Fresh-frozen, irradiated menisci. **b** The bone plug was thinned with scissors. **c** Allograft with bone plug (the bone plug in the black circle is 4 mm in diameter) was marked with radial signs with a surgical marker. **d** Ethibond (No. 5; Ethicon, Somerville, NJ) was placed with a whipstitch at each horn for later traction, spreading, and securing the horn of the allograft

The sizing protocol was based on the 3D CT reconstruction measurements [13] combined with matching of the height, weight and sex between the donor and recipient [14]. In all cases, fresh-frozen, irradiated menisci were used (Fig. 1). All procedures were performed by a single surgeon (Wentao Zhang).

**Fig. 2:**
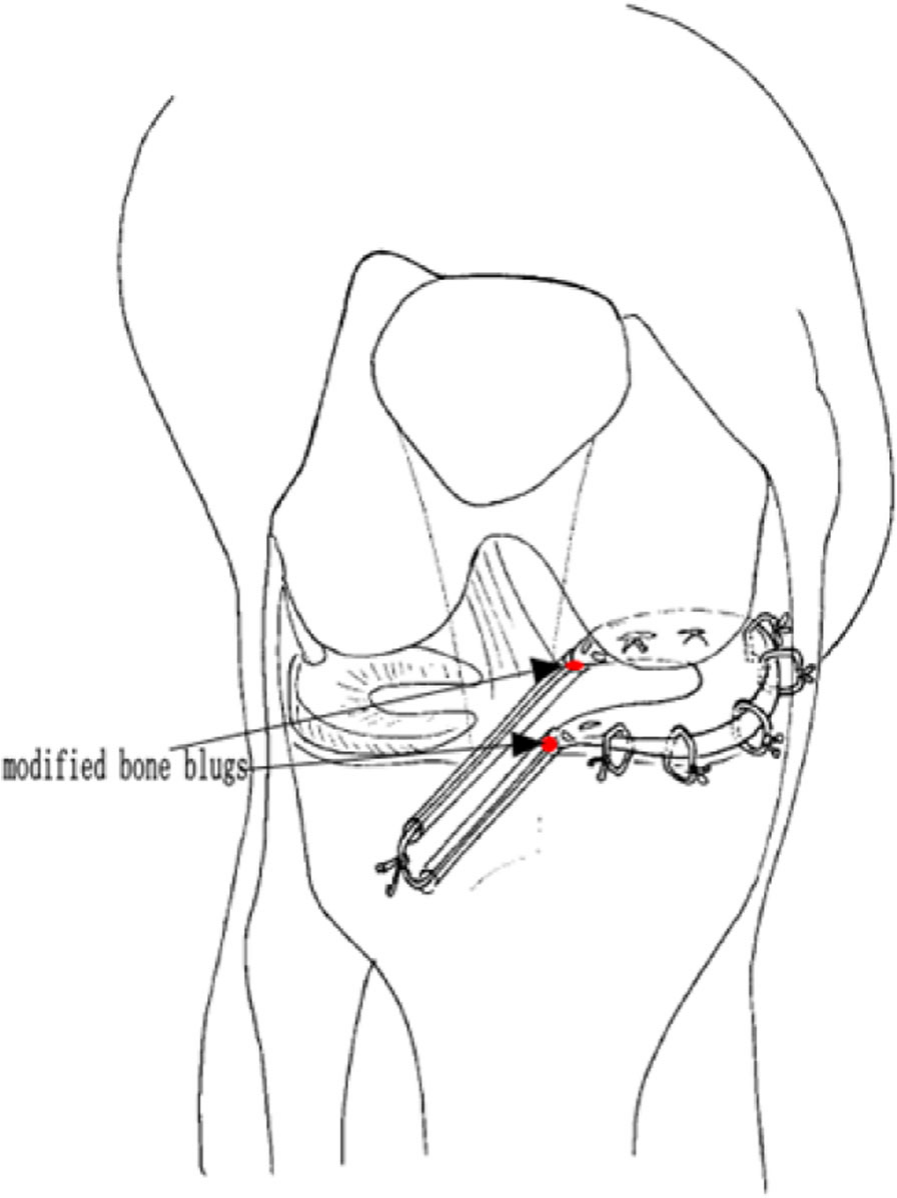
Schematic drawing of the presented arthroscopic modified bone plug technique using double tibial tunnels for meniscal allograft transplantation. Red points mark the bone plugs, and the modified bone plugs were placed in the bone tunnels to permit sound bone-to-bone healing with the tibia

The arthroscopy-assisted MAT was performed with a bone plug technique [15] for MAT. A 4-mm diameter spherical anterior bone plug and a 4-mm diameter spherical posterior bone plug size (approximately the size of a grain of rice) were cored from the meniscal allograft to permit sound bone-to-bone healing with the tibia. Ethibond (No. 5; Ethicon, Somerville, NJ) was placed with a whipstitch in each horn for later traction and spreading. The allograft was marked with radial signs with a surgical marker to prevent mismatching and twisting during arthroscopic insertion. The meniscus remnant was shaved until the meniscus-capsular zone was reached. A tibial guide, a 2.0-mm guide drill and a 4.5-mm core drill were used to prepare two tibial tunnels through which the Ethibonds corresponding to each horn of the menisci were placed. A knot pusher with perforation at one end was used as needed to pass the two Ethibonds through the bone tunnels (Fig. 2).

**Fig. 3:**
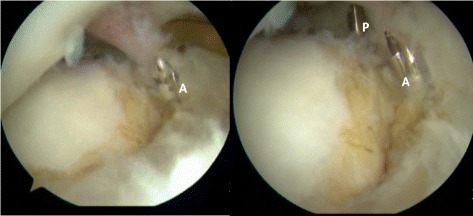
A tibial guide and a 2.0-mm guide drill were used to locate the attachment points of the meniscus posterior horn and anterior horn. A presents the insertion site of the anterior horn of the lateral meniscus, while P presents the insertion site of the posterior horn insertion of the lateral meniscus

Position of bone tunnels: the medial meniscus posterior horn inserts directly anterior to the tibial insertion of the PCL, on the downslope of the posterior intercondylar fossa, behind the posterior horn insertion of the lateral meniscus. The posterior horn of the lateral meniscus inserts directly posterior to the lateral tibial spine, adjacent and anterior to the insertion of the posterior horn of the medial meniscus [16]. The anterior horn of the medial meniscus inserts in line with the medial tibial eminence, approximately 7 mm anterior to the ACL tibial insertion. This insertion site is under the patellar fat pad and is difficult to visualize without debriding a portion of the anterior fat pad. The intermeniscal ligament attaches to the posterior half of this insertion site. The insertion site of the anterior horn of the lateral meniscus is directly anterior to the lateral tibial spine and adjacent to the tibial insertion of the ACL (Fig. 3).

**Fig. 4:**
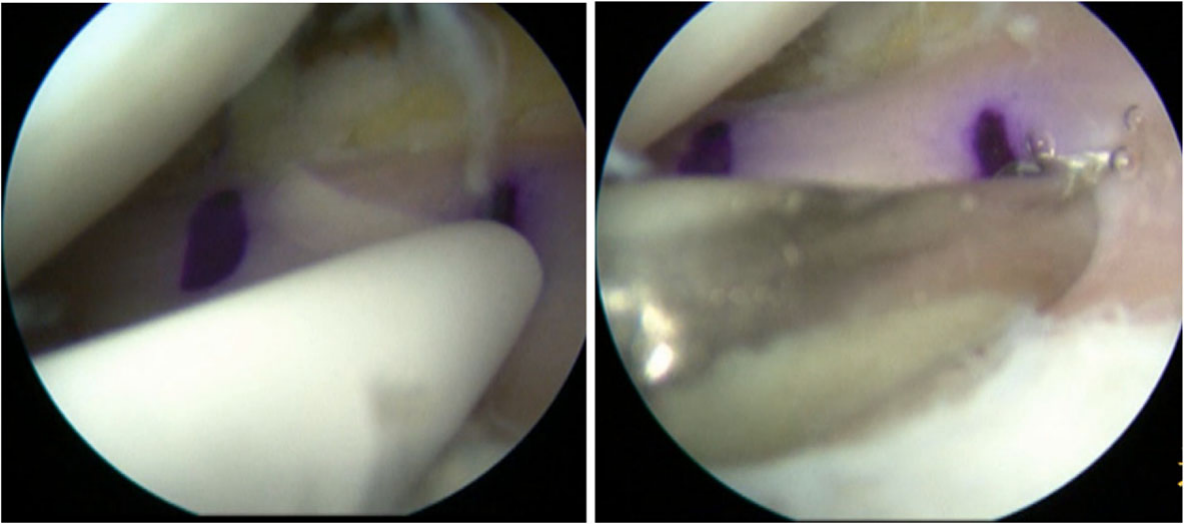
The posterior horn area, which has not received any stitches, is attached to the posterior capsule with Fast-Fix all-inside sutures

**Fig. 5:**
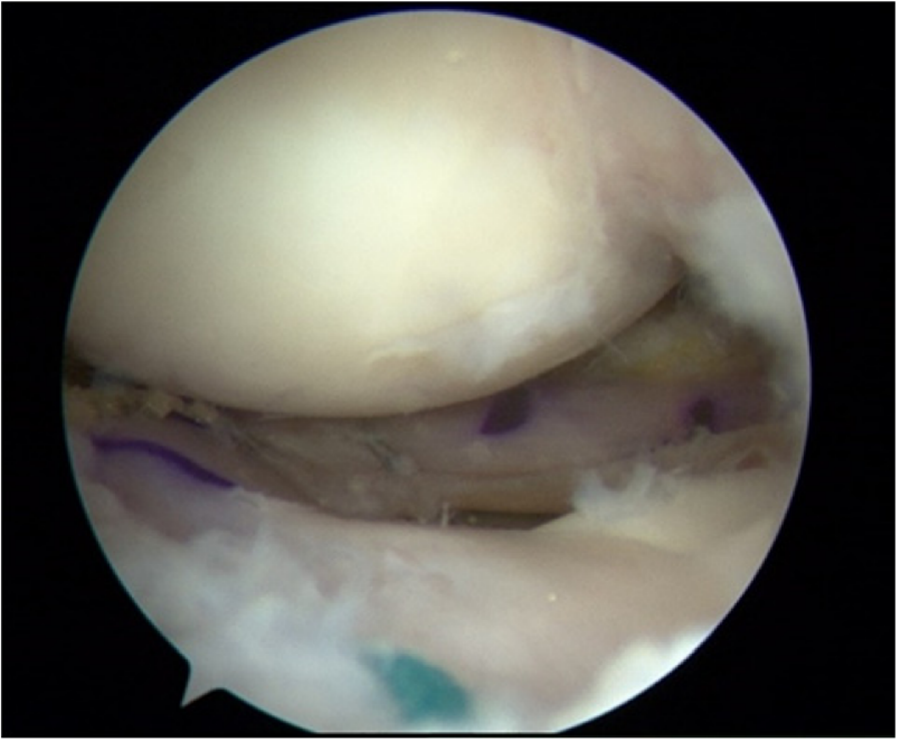
Final arthroscopic view of an implanted medial meniscal allograft in the left knee of a female 35-year-old patient

Suturing was started from posterior to anterior in order to firmly attach the graft to the joint capsule. All sutures from the body of the meniscus were tied to the capsule. The posterior horn area, which did not receive any stitch, was attached to the posterior capsule with Fast-Fix (S&N, Andover, MA, USA) all-inside sutures (Fig. 4). Before suturing, the correct placement of the graft was checked. The lower suture should be removed before the upper one. When all sutures are outside the knee, they can be sutured to the capsule. If additional stitches are necessary to ensure a stable graft, this stitch must be out-in. Finally, the sutures from the anterior and posterior tunnels were sutured to each other, leaving both meniscus horns anchored [8]. Then, the transplanted meniscus was checked for stability and matching (Fig. 5).

The mean time of the operation was 2.05 h. Of the transplants, 13 were performed concomitant with ACL reconstruction.

### Rehabilitation

Early ROM for 15 min daily in the range of 0° to 60° was encouraged beginning 1 week postoperatively to minimize the deleterious effects of immobilization. Gradual progression to full weight bearing occurred by 6 weeks postoperatively. A double upright hinged brace was used during this designated protection phase. Flexion was limited to 90 for the first 6 weeks, as progressive knee flexion subjects the meniscus to greater stress [9]. Full ROM was achieved by 14 weeks postoperatively. Gentle jogging could begin between 4 and 6 months, but running was not advised before 6 months post-surgery. Finally, return to sports was anticipated to occur between 6 and 9 months [7]. In cases of concomitant ACL reconstruction, the rehabilitation protocol was modified according to the surgeon’s concern for both the ACL and the meniscus. Training to strengthen muscles and improve proprioception was conducted throughout the rehabilitation protocol.

### Follow-up

All patients were followed up for more than 18 months postoperatively for ROM, International Knee Documentation Committee (IKDC) score, Lysholm score, Tegner score, and VAS for pain. Physical examinations and associated measurements were repeated postoperatively. Physical examination and radiographic parameters during follow-up were available in all cases. Patients were asked two series of questions: 1. Were you able to return to your previous level of activity? (Yes/No). If yes, what sports could you do? Can you do a single-leg jump? If no, what degree from 0 to 100% according to the visual analogue scale after surgery did you reach compared with your previous level of activity and what is the main factor limiting the activity? 2. Based on your experience, would you have the motivation to seek the same treatment in case of being subjected to the same injury on the contralateral knee? (Yes/No/Unsure). All responses were collected and recorded by a single observer. The intraobserver reliability of this observer ranged from moderate to excellent for all parameters tested. We evaluated cartilage status according to the modified Outerbridge classification system. MRI examinations were performed on a 3 T cylinder-shaped instrument. Orthopedic surgeons measured meniscal extrusion to the nearest millimeter on the coronal images and obtained mean values and standard deviation. The relative percentage of meniscal extrusion was defined as the percentage of extruded meniscal width compared with the entire meniscal width [17].

### Statistical analysis

Statistical analysis was performed with SPSS software for Windows (version 19.0, SPSS Inc., Chicago, IL, USA). The data were tested via the normality test (Shapiro–Wilk test, *n* < 2000) and did not follow a Gaussian distribution. The Wilcoxon rank sum test was used to compare preoperative and postoperative IKDC score, Lysholm score, Tegner score, VAS, cartilage status and to correlate the influence of associated procedures and previous lesions. Data on meniscal extrusion and RPE were analyzed. Spearman correlation analysis was used to determine whether the changes in MAT follow-up results were associated with possible risk factors, including age at time of MAT, meniscal extrusion, the time from the total meniscectomy to the secondary MAT. *P* < 0.05 was considered significant.

## Results

Cartilage status was evaluated by MRI according to the modified Outerbridge system. The mean meniscal extrusion was 3.39 ± 0.90 mm, the relative percentage of extrusion (RPE) was 34.82 ± 12.71%, and arthrosis progression was observed in 8 of 61 cases (13.1%), 6 of which also underwent ACLR. The Wilcoxon rank sum test showed there was no significant difference between preoperative and postoperative cartilage status (Table 2), and there was no significant difference in MAT follow-up results between different preoperative cartilage status groups (Table 3). We found significant association between the changes in postoperative IKDC score (positive correlation, *P* < 0.05), Lysholm score (positive correlation, *P* < 0.05), Tegner score (positive correlation, *P* < 0.05), VAS (negative correlation, *P* < 0.05), and the time from the total meniscectomy to the secondary MAT and meniscal extrusion (*P* < 0.05), no significant association between the changes in MAT follow-up results and age (*P* > 0.05) (Table 4).

**Table 2:** Cartilage status on MRI

Arthrosis grade on MRI	Preoperatively	Postoperatively
0	8	7
1	17	13
2	23	25
3	7	8
4	6	8
*P* Value	*Z* = − 0.911	*P* = 0.36

**Table 3:** Comparisons of MAT follow-up results between different preoperative cartilage status groups (Wilcoxon test)

Arthrosis grade on MRI (preoperatively)	*N*	Post-op VAS	Post-op IKDC	Post-op Tegner	Post-op Lysholm
		P_50_ (P_25_–P_75_)	P_50_ (P_25_–P_75_)	P_50_ (P_25_–P_75_)	P_50_ (P_25_–P_75_)
0	8	4 (3.25, 5.75)	78 (70.5, 87)	4.5 (4, 5.75)	81.5 (76.5, 85)
1	17	3 (3, 4)	82 (79, 85.5)	6 (5, 6)	83 (82, 86.5)
2	23	3 (3, 4)	84 (81, 87)	6 (5, 6)	87 (84, 89)
3	7	3.5 (3, 4)	85 (80, 88)	5 (5, 6)	85 (75, 89)
4	6	4 (2.75, 5)	85 (82, 86.75)	5.5 (5, 6)	85.5 (83, 88.75)
H		*H* = 5.4, *p* = 0.249	*H* = 4.6, *P* = 0.331	*H* = 7.746, *P* = 0.101	*H* = 7.56, *P* = 0.109

*P50* median, *P75–P25* interquartile range)

**Table 4:** The changes in MAT follow-up results associated with possible risk factor (Spearman correlation analysis)

	Post-op VAS	Post-op IKDC	Post-op Tegner	Post-op Lysholm
Age	Pearson = 0.03, *P* = 0.821	Pearson = −0.034, *P* = 0.798	Pearson = −0.054, *P* = 0.678	Pearson = −0.028, *P* = 0.831
The time from the total meniscectomy to the secondary MAT	Pearson = −0.342, *P* = 0.08	Pearson = 0.559, *P* = 0.002	Pearson = 0.467, *P* = 0.014	Pearson = 0.565, *P* = 0.002
Meniscal extrusion	Pearson = 0.323, *P* = 0.011	Pearson = −0.286, *P* = 0.026	Pearson = −0.235, *P* = 0.068	Pearson = − 0.263, *P* = 0.041

The mean results for postoperative VAS, IKDC score, and Lysholm score were significantly better than the data for preoperation (*P* < 0.05, Wilcoxon test), while there was no significant difference in the ROM and Tegner score pre- and postoperation (*P* > 0.05, Wilcoxon test) (Table 5).

**Table 5:** Comparisons of preoperative and postoperative data in all 61 cases (Wilcoxon test)

Items	N	Pre-op		Follow-up		W	*P*
		P_50_	P_75_–P_25_	P_50_	P_75_–P_25_		
VAS	61	5	3	3	1	1478.5	< 0.001
IKDC	61	68	15	84	6	146	< 0.001
Tegner	61	5	2	6	1	467	0.062
Lysholm	61	69	13	85	5	141	< 0.001
ROM	61	145	10	145	10	709	0.122

*P50* median, *P75–P25* interquartile range)

Comparing the outcome scores between lateral and medial transplantation revealed no significant difference in (ROM), IKDC score, Lysholm score, Tegner score, or VAS (*P* > 0.05, Wilcoxon test) (Table 6).

**Table 6:** Comparison of outcome scores between lateral and medial transplantation (Wilcoxon test)

Items	Medial MAT (*N* = 6) Follow-up–pre-op		Lateral MAT(*N* = 52) Follow-up–pre-op		W	*P*
	P_50_	P_75_–P_25_	P_50_	P_75_–P_25_		
VAS	− 2	2	− 2	2	202.5	0.521
IKDC	21	7	13.5	14.75	313.5	0.108
Tegner	1	1	0	2.25	303.5	0.154
Lysholm	22	10	15	10.75	310	0.124
ROM	0	15	0	16.25	265	0.529

*P50* median, *P75–P25* interquartile range)

Comparing the outcome scores between MAT, MAT+MF, and MAT+ACLR revealed no significant difference in IKDC score, Lysholm score, Tegner score, or VAS (*P* > 0.05, Wilcoxon test) (Table 7). Only 3 patients with MAT+MF + ACLR, 2 with OT, 1 with MCLR, and 1 with open reduction internal fixation (ORIF), there was no reliable statistical analysis to correlate the influence of these associated procedures because of smaller number.

**Table 7:** Comparisons of MAT follow-up results between different associated surgery procedures (MAT, MAT+MF, and MAT+ACLR) (Wilcoxon test)

Procedure	N	Post-op VAS	Post-op IKDC	Post-op Tegner	Post-op Lysholm
		P_50_ (P_25_–P_75_)	P_50_ (P_25_–P_75_)	P_50_ (P_25_–P_75_)	P_50_ (P_25_–P_75_)
MAT	38	3.5 (3, 4)	84 (79.75, 86.25)	6 (5, 6)	85 (82, 87)
ACL+MAT	10	3.5 (3, 5)	80 (76, 85.5)	5.5 (4, 6.25)	84 (75.5, 90)
MF+MAT	8	3 (2.75, 3.25)	85 (79.75, 88)	5.5 (5, 6)	84.5 (77.25, 89.25)
*H*		*H* = 3.378, *P* = 0.185	*H* = 2.436, *P* = 0.296	*H* = 0.517, *P* = 0.772	*H* = 0.079, *P* = 0.961

**Fig. 6:**
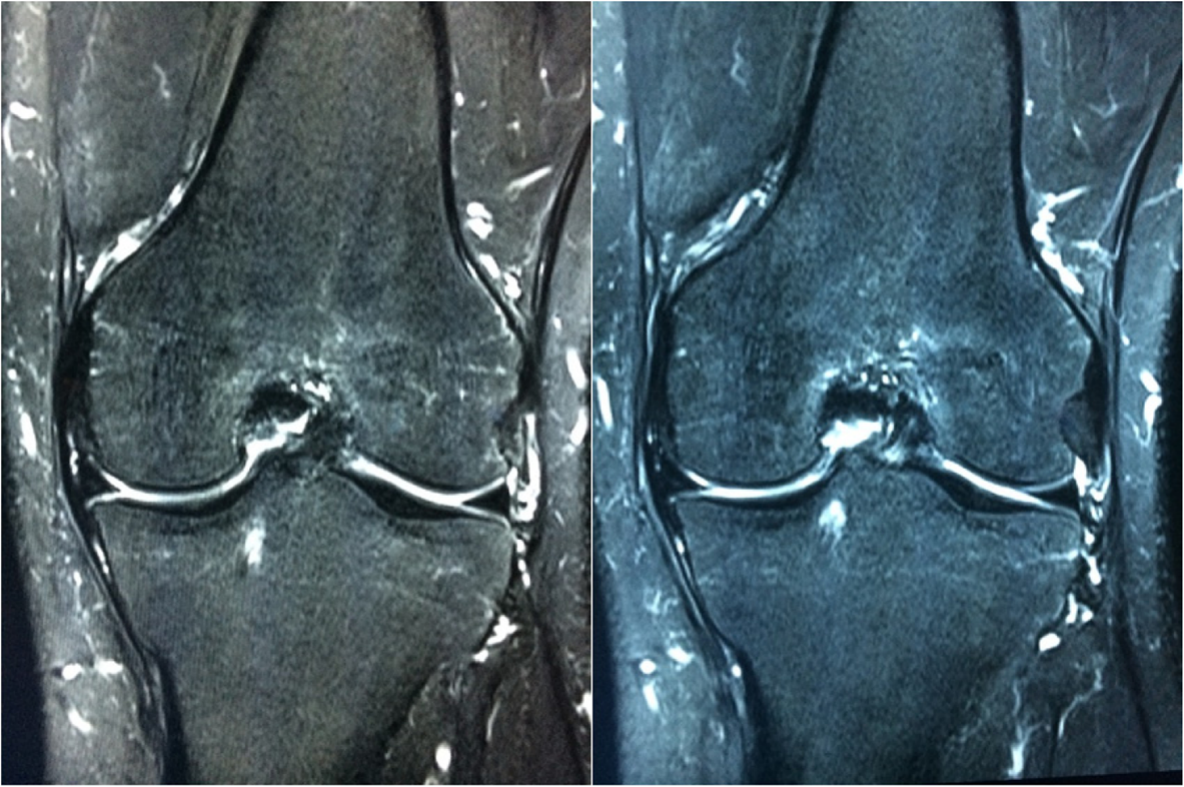
The modified bone plugs were inserted into the bone tunnels to achieve sound bone-to-bone healing with the tibia 2 years after MAT

With regard to the postoperative examination of the patients who underwent MAT combined with ACL reconstruction, the anterior drawer test and Lachman test were negative. All X-rays and MRI of the 51 patients showed no joint space narrowing. According to the Stoller standard, with regard to reinjury of the transplanted meniscus, 13 showed no reinjury, 25 I° reinjury, 17 II° reinjury and 6 III° reinjury at the 18-month follow-up. All of the meniscus formed a union with the capsule confirmed by MRI. The modified bone plugs were inserted into the bone tunnels to form sound bone-to-bone healing with the tibia 2 years after meniscal allograft transplantation (Fig. 6). One incision showed delayed union combined with fat liquefaction. Two patients who underwent concomitant ACL reconstruction developed synarthrophysis. One suffered a postoperative joint infection. There was one failure of the operation and one meniscus allograft dislocation, treated with a second operation for meniscal restoration.

Thirty-eight (62.3%) patients were able to return to their previous level of activity, and 25 of them returned to football or basketball, 2 returned to tennis, 3 returned to table tennis, 5 returned to badminton, and 3 returned to Kung fu; however, in 3 of these cases, the medial side of the knee was slightly painful during a single-leg jump. The remaining 23 patients reached a mean 76.7% of the previous level of activity. Of the 23 (37.7%) patients reporting a decrease in activity, 10 reported the fear of reinjury as the primary factor limiting activity. Of the remaining patients, pain (*n* = 5), limitation of ROM (*n* = 4), medical advice (*n* = 3), and a change in life situation (*n* = 1) were the reported primary reasons for decreased activity.

Asked if they would choose to undergo the procedure again in case of being subjected to the same injury on the contralateral knee based on their experience, 42 (68.9%) patients stated that they would, 4 cases (6.5%) said they would refuse the possibility of the same treatment, and 15 (24.6%) were unsure about their decision.

## Discussion

During this research, cartilage status was evaluated by MRI according to the modified Outerbridge system, and the Wilcoxon rank sum test showed there was no significant difference between preoperative and postoperative cartilage status. Arthrosis progression was observed in 8 of 61 cases (13.1%). Among these patients, 6 cases were reinjured after MAT, and there was one failure of the operation and one meniscus allograft dislocation, and there was no significant difference in MAT follow-up results between different preoperative cartilage status groups. It has been speculated that MAT with the new modified bone plug technique with anatomical meniscal root reinsertion could delay the progression of arthrosis. This conclusion is in accordance with Bum-Sik Lee’s result—“Articular cartilage degenerates after subtotal/total lateral meniscectomy but radiographic arthrosis progression is reduced after meniscal transplantation” [18]—and is in line with prior research using a bone plug technique [17, 19]. In addition, there was significant association between MAT follow-up results and the time from the total meniscectomy to the secondary MAT, when the time from the previous meniscectomy to MAT increased, the follow-up results of MAT got worse.

Wang et al. [20] found that transosseous fixation at the meniscal horns provides superior load distribution in the involved knee compartment after meniscal transplantation compared with suture-only fixation. To a certain extent, this finding showed our modified technique was reliable. However, unlike traditional bone plug or bone bridge techniques, the modified technique requires little space to fix the anterior or posterior horns of the meniscus and does not damage the tibial insertion of the PCL and ACL. Almost all of the allografts demonstrated sound bone-to-bone healing with the tibia 2 years after MAT, in contrast to Roberson’s soft tissue in bone socket fixation technique [21].

The mean meniscal extrusion was 3.39 ± 0.90 mm, and the relative percentage of extrusion (RPE) was 34.82% ± 12.71% in our study, which is slightly superior to the traditional bone plug technique, whose mean meniscal extrusion and the relative percentage of extrusion were reported to be 4.35 ± 1.76 mm and 43% ± 19.8%, respectively [19]. This difference might be due to racial differences or technique, but there is not enough evidence to be sure. And we found significant association between the changes in MAT follow-up results and meniscal extrusion, and it was different from Ji Hyun Ahn’s [22] that there were no significant difference in Lysholm score and Tegner activity scale between minor extrusion group and major extrusion group after MAT.

In our patients, including cases with additional orthopedic procedures, MATs were performed simultaneously or in a staged manner along with ligament surgery such as ACLR or MF occurred most frequently. We conducted a subgroup analysis of both isolated MAT, MAT procedures with concomitant ligament surgery, and MAT with MF. And there were no significant difference in them at minimum 18-month follow-up, and the results were in accordance with Bum-Sik Lee [23]. With only three patients with MAT+MF+ACLR, two with OT, one with MCLR, and one with ORIF, there was no reliable statistical analysis to correlate the influence of these associated procedures because of the smaller number.

In addition, of the 23 (37.7%) patients reporting a decrease in activity, 10 patients reported fear of reinjury as the primary factor limiting activity. We found in many cases that excessive flexion was limited compared with that of the contralateral knee. However, rehabilitation of the last angles of excessive flexion is somewhat difficult and painful, and most patients accepted the 5° limitation and refused to continue the rehabilitation. A systematic review reported that MAT allows return to the same level of competition in 75–85% of patients in the short- to mid-term follow-up [24], the same as in our results. A recent systematic review [25] reported that 70–92% of patients returned to a wide variety of sports activities, and there was no association between mean Tegner scores and transplant failure rates, but a moderate correlation was found between failure rates and mean follow-up time (*R* = 0.63). An analysis of the effect of return to high-impact activities on transplant failure rates or progression of OA was not possible because of the short-term follow-up.

Patients who do not resume sports primarily cannot return to their previous sports for two reasons: first, the allograft may prevent certain motions, and second, the rehabilitation protocol may have been conducted poorly. The use of a rehabilitation protocol that is appropriate for the surgical technique results in optimal postoperative outcomes and functional recovery of patients to a preinjury level of activity. The ideal rehabilitation program is based on biological and mechanical knowledge of the meniscus. Rehabilitation programs following meniscal transplantation are dependent on surgical technique, concomitant procedures, and pathology as well as surgeons’ preference [26]. Rehabilitation protocols must strike a balance between maintaining joint motion and muscle strength and protecting the graft to allow full healing of the meniscus and any associated procedures [27]. To achieve this, most authors limit weight bearing and range of motion for up to 6 weeks before progressively allowing the resumption of activities. Rehabilitation protocols are divided into “traditional”, involving restricted range of motion, weight bearing and improving muscle strength, and “aggressive”, involving an intensive physiotherapy program, especially kinesiotherapy (achieving full extension immediately after surgery and early implementation of therapy combining concentric and eccentric contraction for the thigh muscle and exercise in the open kinetic chain). Our rehabilitation protocol belongs to the group of “traditional” rehabilitation protocols.

To the best of our knowledge, our study is the largest to report the outcomes of MAT using a modified bone plug technique with anatomical meniscal root reinsertion in China, and this study is unique. In this series, we have shown that the majority of patients return to their preinjury level of sports after this procedure. As with most procedures, the patients must be made fully aware of the uncertain effect of MAT on the eventual progression of articular cartilage degeneration and deterioration or traumatic disruption of their meniscus grafts. Longer term of follow-up should be continued to provide additional information.

This study has certain limitations. First, further details regarding the specific type and competitive level of pre- and postoperative sport and activity for each patient are not available for this cohort. Second, as a successful return to play may take up to 2 years after surgery, the follow-up should exclude those with less than 2 years following surgery. Finally, it is difficult to draw any final and definite conclusions. Despite these limitations, this retrospective cohort provides updated results regarding a new MAT technique in China.

## Conclusion

Our modified bone plug method with anatomical meniscal root reinsertion is an effective surgical method, and the majority of active patients with meniscal disorders returned to preinjury levels of activity.

## Abbreviations

ACL: Anterior cruciate ligament
ACLR: Anterior cruciate ligament reconstruction
CMSK: Contralateral meniscus in the same knee
IKDC: International Knee Documentation Committee
L/M-MAT: Lateral and medial MAT concomitantly
L-MAT: Lateral MAT
MAT: Meniscal allograft transplantation
MCLR: Medial collateral ligament reconstruction
Microfx: Microfracture
M-MAT: Medial MAT
MRI: Magnetic resonance imaging
ORIF: Open reduction internal fixation
OT: Chondral transplantation
PM: Partial meniscectomy
RPE: Relative percentage of extrusion
VAS: Visual analogue scale
